# Supplementary material for: BPA Exposure Affects Mouse Gastruloids Axial Elongation by Perturbing the Wnt/β-Catenin Pathway
Source: Int J Mol Sci. 2024 Jul 19;25(14):7924. doi: 10.3390/ijms25147924 (PMC11276681; doi:10.3390/ijms25147924)
Supplement: Supplementary file 1 [file ijms-25-07924-s001.zip › ijms-3077523-supplementary.pdf]

Table S1. Reverse and forward primer sequences used for qRT-PCR.

Gene	Forward primers	Reverse primers
Snail	5′ AGTTGACTACCGACCTTG 3′	5′ AAGGTGAACTCCACACAC 3′
Slug	5′ GACACATTAGAACTCACACTG 3′	5′ GACATTCTGGAGAAGGTTTTG 3′
Twist	5′ GAGACCTAGATGTCATTGTTTC 3′	5′ GAATTTGGTCTCTGCTCTTC 3′
β2M	5′ GAATTCACCCCCACTGAGACT 3′	5′ TGCTTGATCACATGTCTCGAT 3′
